# Research in abdominal imaging – current status and trends at ECR 2020

**DOI:** 10.1007/s00330-020-06936-0

**Published:** 2020-05-20

**Authors:** Thomas C. Lauenstein

**Affiliations:** Department of Radiology, Evangelisches Krankenhaus Düsseldorf (EVK), Kirchfeldstrasse 40, 40217 Düsseldorf, Germany

During the last decades, diagnostic radiology in general and abdominal imaging in particular have gone through substantial changes and developments. Hereby, a shift of paradigm could be observed from mere diagnostic assessment of pathological disorders to the evaluation (and even prediction) of therapeutic success based on imaging parameters. Furthermore, a quantitative tissue analysis of different organs based on multi-parametric imaging has become feasible. At the European Congress of Radiology (ECR) 2020, the latest research in abdominal imaging was presented in a total of seven scientific sessions. This editorial does not aim to give an all-encompassing summary of all scientific lectures. Rather, a subjective selection of highlights is presented with a focus on three main topics of abdominal imaging including (a) pancreatic imaging, (b) prediction of therapeutic success based on imaging features and (c) advances in order to optimize and shorten imaging protocols.

- Pancreatic imaging

Pancreatic cystic lesions (PCL) as incidental findings play a huge role in the daily clinical practice. Although the majority of these lesions only has a low risk of malignant transformation, there are several aspects that remain unsolved including (a) if and when endoscopic or surgical intervention is needed, (b) worrisome imaging features of cystic lesions that may be considered markers for an increased risk of malignancy and (c) the time interval for imaging surveillance of patients with cystic pancreatic lesions. Zhu et al evaluated MR morphologic features as well as clinical data predicting the progression of PCL [1]. More than 2.000 MR scans with PCL were retrospectively analyzed. Overall, 37.7% of PCL progressed during follow-up. Imaging or clinical features that were independently associated with lesion growths included patients’ age, number of lesions, communication with the pancreatic duct and presence of septa within the cyst. In only a minority of patients with growing PCL (27.4%) an intervention was needed as lesions showed worrisome features or increased in size > 30 mm. Hence, this data of a large retrospective cohort may help us how to deal with PCL in the future.

Boraschi et al performed a study in a similar context [2]. They aimed to assess the safety of a surveillance MR protocol in patients with branch-duct intraductal papillary mucinous neoplasm (BD-IPMN). Sixty-nine patients with BD-IPMN were included and underwent MR scans over a time range of 10 years. The surveillance protocol included MR scans every 6 months during the first two years and on a yearly basis thereafter. In this trial worrisome features developed in 14.5% and only 7% of patients finally underwent surgery. Overall, cysts only increased slowly in size with an average annual growth rate of 1 mm.

These studies carry several messages I believe to be important: cystic pancreatic lesions only show a slow increase in size and therefore are suitable for imaging surveillance. Only a minority of patients has yet to undergo surgery. However, radiologists should be aware of risk factors that may lead to a change of the surveillance rate. These factors include patients’ age, number of lesions, presence of septa and visible communication with the pancreatic duct.

(b) Prediction of therapeutic success based on imaging features

One of the most challenging questions particularly in the setting of cancer imaging remains the prediction of therapy success for different therapeutic strategies. Wei et al utilized gadoxetic acid-enhanced MRI in order to identify preoperative imaging biomarkers for the prediction of tumor recurrence in patients with HCC [3]. A total of 126 subjects with 167 HCC lesions were imaged within one month prior to tumor resection. Almost half of the patients (n = 62) developed a recurrent tumor within 24 months after surgery. Four independent features were found to correlate with the risk of tumor recurrence including (a) peritumoral low signal intensity on the hepatobiliary phase, (b) corona enhancement, (c) tumor size > 5 cm and (d) multifocal tumor disease. In another study by Vosshenrich et al, 37 patients with 92 HCC lesions were enrolled [4]. CT scans were performed before and after transarterial chemoembolization (TACE). Quantitative CT texture parameters were extracted including long axis of tumor, mean of positive pixels and uniformity of positive pixel distribution. All these features were found to be good discriminators for the prediction of complete response vs. progressive disease. Authors conclude that a multiparametric model based on CT texture analysis could serve as a valuable tool when triaging patients with HCC to TACE.

Overall, these trials underline the great potential of multiparametric imaging in tumor patients with vast clinical consequences. Imaging features may help to answer the question whether a specific treatment may be successful or if other alternatives should be taken into consideration. Furthermore, it may be a guideline to tailor surveillance strategies: if pretherapeutic imaging reveals high risk of tumor recurrence, time intervals for follow-up imaging need to be adapted. Despite these promising results, there are still several open questions. One challenge is related to the practicability of these strategies. What soft- and hardware is needed and how time-consuming is data evaluation? Furthermore, a large variety of studies have been performed so far at different centers using different imaging modalities and different imaging parameters. Hence, it would be advantageous to synchronize results in order to allow for a more uniform implementation.

(c) Advances in order to optimize and shorten imaging protocols

All-encompassing MR examinations of the abdomen are often time consuming due to the large number of sequences that are acquired. Examination slots yet are limited and valuable. Thus, one may wonder whether we really need to perform extended MRI examinations all the time or if it is possible to use shortened protocols that are adapted to the clinical situation. This may be particularly interesting when high risk patients undergo screening procedures. Pecorelli et al focused on this issue analyzing patients with liver cirrhosis or chronic hepatitis B, who have an increased risk for the development of hepatocellular carcinoma (HCC) [5]. An MRI protocol including only three sequences (T2w single shot fast spin echo, diffusion weighted imaging and hepato-biliary phase T1w MRI) was used resulting in a total in-room time of less than 15 minutes. A total of 376 patients were analyzed with clinical, imaging and pathological data serving as the standard of reference. Sensitivity, specificity and accuracy of this abbreviated MRI protocol amounted to 86%, 91%, and 90% respectively. Hence, authors concluded that this surveillance protocol for HCC detection is feasible providing high diagnostic accuracy.

A similar study was performed by Willemssen et al [6]. They examined 215 patients at risk for HCC. The abbreviated MRI protocol as well included three sequence types (T2w imaging, diffusion weighted imaging (DWI) and T1w in and opposed phase imaging). Reference standard was a conventional extended MRI protocol for liver imaging. Thirty-seven of 39 HCC were detected by means of the short MRI protocol and only two HCC lesions were missed (sensitivity: 94.9%). Hence, the protocol utilized in this trial can be considered a promising technique for surveillance of HCC without the need of i.v. contrast, which may be another beneficial aspect.

The evaluation of short MRI protocols is promising and will encourage radiologists to tailor examinations to the clinical circumstances. Hence, a dedicated first time MRI exam of the liver could encompass more or different sequences than a follow-up exam or a surveillance exam of patients with a high-risk profile. If we follow this strategy, examination time can be spared and more exams per time unit could be performed. However, there are still some problems to be solved, e.g., when to use i.v. contrast. Furthermore, the proposals for short MRI protocols are somewhat heterogeneous. It will be the task of expert groups or multi-center trials to overcome this limitation in the future.
